# Assessment of the impact of different fecal storage protocols on the microbiota diversity and composition: a pilot study

**DOI:** 10.1186/s12866-019-1519-2

**Published:** 2019-06-28

**Authors:** Shirin Moossavi, Phillip A. Engen, Reza Ghanbari, Stefan J. Green, Ankur Naqib, Faraz Bishehsari, Shahin Merat, Hossein Poustchi, Ali Keshavarzian, Reza Malekzadeh

**Affiliations:** 1 Digestive Oncology Research Center, Digestive Disease Research Institute, Tehran University of Medical Sciences, Tehran, Iran; 2 Department of Medical Microbiology and Infectious Diseases, University of Manitoba, Winnipeg, MB Canada; 3 Department of Internal Medicine, Division of Gastroenterology, Rush University Medical Center, Chicago, IL USA; 4 Department of Nutrition, Nutrition Research Institute, University of North Carolina at Chapel Hill, Chapel Hill, NC USA; 5 Sequencing Core, Research Resources Center, University of Illinois at Chicago, Chicago, IL USA; 6 Department of Biological Sciences, University of Illinois at Chicago, Chicago, IL USA; 7 Liver and Pancreatobiliary Diseases Research Center, Digestive Disease Research Institute, Tehran University of Medical Sciences, Shariati Hospital, Kargar Shomali Avenue, Tehran, Iran; 8 Department of Pharmacology, Rush University Medical Center, Chicago, IL USA; 9 Department of Physiology, Rush University Medical Center, Chicago, IL USA; 10 Division of Pharmacology, Utrecht Institute for Pharmaceutical Sciences, Utrecht University, Utrecht, Netherlands

**Keywords:** Gut microbiota, Cohort study, Fecal storage, Card, Mock shipment

## Abstract

**Background:**

Fecal samples are currently the most commonly studied proxy for gut microbiota. The gold standard of sample handling and storage for microbiota analysis is maintaining the cold chain during sample transfer and immediate storage at − 80 °C. Gut microbiota studies in large-scale, population-based cohorts require a feasible sample collection protocol. We compared the effect of three different storage methods and mock shipment: immediate freezing at − 80 °C, in 95% ethanol stored at room temperature (RT) for 48 h, and on blood collection card stored at RT for 48 h, on the measured composition of fecal microbiota of eight healthy, female volunteers by sequencing the V4 region of the 16S rRNA gene on an Illumina MiSeq.

**Results:**

Shared operational taxonomic units (OTUs) between different methods were 68 and 3% for OTUs > 0.01 and < 0.01% mean relative abundance within each group, respectively. α and β-diversity measures were not significantly impacted by different storage methods. With the exception of Actinobacteria, fecal microbiota profiles at the phylum level were not significantly affected by the storage method. Actinobacteria was significantly higher in samples collected on card compared to immediate freezing (1.6 ± 1.1% vs. 0.4 ± 0.2%, *p* = 0.005) mainly driven by expansion of Actinobacteria relative abundance in fecal samples stored on card in two individuals. There was no statistically significant difference at lower taxonomic levels tested.

**Conclusion:**

Consistent results of the microbiota composition and structure for different storage methods were observed. Fecal collection on card could be a suitable alternative to immediate freezing for fecal microbiota analysis using 16S rRNA gene amplicon sequencing.

**Electronic supplementary material:**

The online version of this article (10.1186/s12866-019-1519-2) contains supplementary material, which is available to authorized users.

## Background

Gut microbiota bridges the interaction of host and environment and is centrally located in the mechanistic framework of multiple complex intestinal and extraintestinal diseases with multifactorial aetiologies such as inflammatory bowel disease and cardiovascular disease [1]. Accordingly, gut microbiota characterization is an important pillar of precision medicine [2] and is investigated in population-based cohorts [3–5]. Analysis of fecal samples is frequently used as a surrogate for studying the gut microbiota with immediate freezing being the gold standard method for preserving the microbial community profile [6]. However, this method introduces logistical difficulties and may not be feasible for all large-scale population-based cohort studies especially in rural and remote settings [7].

Appropriate fecal sampling methods and storage conditions are essential to avoid distorting the original microbial community profile and to minimize the levels of introduced biases in microbiota assessment [6, 8]. Although immediate freezing is commonly considered the gold standard, this approach is not always possible for home sample collection, collection from remote areas, or in large-scale, population-based studies [7]. A variety of alternative methods of sample collection have been proposed in cohort studies, such as the use of preservatives (e.g.*,* ethanol or other solutions) [9–11] or collection on fecal occult blood test (FOBT) cards, which are commonly used for fecal collection for colorectal cancer screening [12]. Furthermore, the impact of delayed freezing has been assessed by simulating mock shipments at ambient temperature [13, 14]. These methods inhibit the continued microbial growth after defecation and, thereby preserve the in vivo microbial community composition [7]. Previous studies have detected an overall high agreement between alternative fecal collection/storage methods compared to immediate freezing [13–17]. Sample storage in ethanol or card could be ideal methods for population-based cohorts given their relatively low cost and/or storage volume. Therefore, our objective was to assess different storage methods and mock shipment of fecal sample in terms of the microbial community structure in a pilot study in healthy female volunteers to identify a feasible method for large scale sample collection in our population-based cohorts.

## Results

**Fig. 1:**
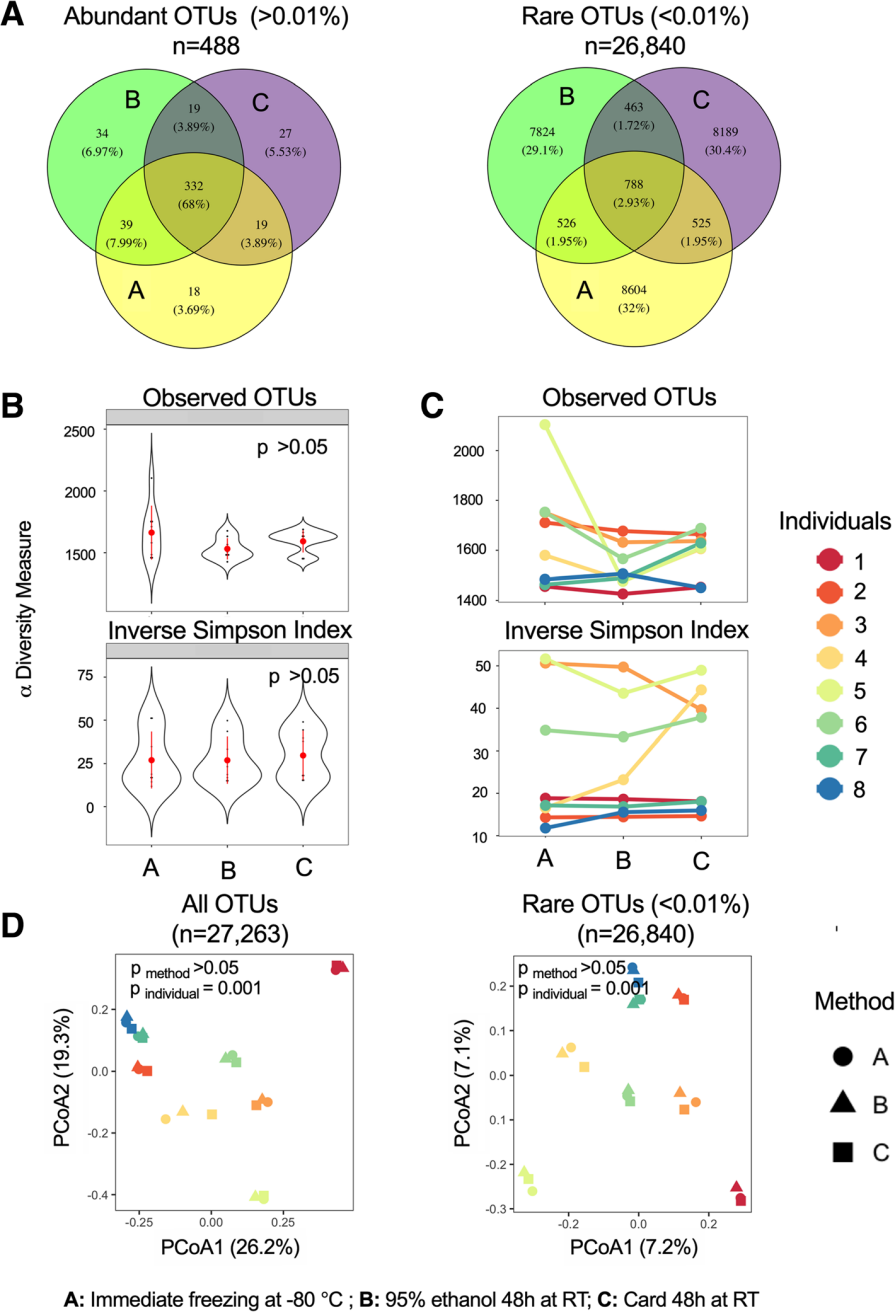
Effect of storage condition on the fecal microbiota profile. **a** Venn diagram of abundant and rare operational taxonomic units (OTUs) defined as having mean relative abundance of > 0.01% and < 0.01% within each method, respectively. **b** α diversity across methods, and **c** across individuals. **d** β diversity based on Bray-Curtis dissimilarity for all and rare OTUs (mean relative abundance < 0.01%)

After merging, trimming and quality control, the sequencing yielded total counts of 1,089,909 sequences with a mean of 45,412 (SD = 12,728) per sample. In all samples from healthy female participants, sequences derived from the phyla Firmicutes and Bacteroidetes dominated in all individuals and treatments. At finer taxonomic resolution, 488 operational taxonomic units (OTUs) were identified to have mean relative abundances of at least 0.01% in one of the methods (immediate freezing *n* = 408, ethanol *n* = 424, and card *n* = 397) with only 68% of them being shared in all methods. The shared proportion of OTUs was 3% for rare OTUs (< 0.01% within each group) (Fig. 1a). Approximately 5% of abundant OTUs were unique to each storage condition (Fig. 1a). Similar results were observed for abundant species and only 37% of all species were common to different methods (Additional file 1: Figure S1).

Alpha diversity was assessed using observed OTUs and the inverse Simpson index on the rarefied data. α diversity metrics were not significantly different based on the method of storage (Fig. 1b). However, within each individual, the measured α diversity metrics differed between sample storage treatments. The difference was more pronounced for the number of observed OTUs compared to inverse Simpson index. While some samples had consistent α diversity across different storage methods (e.g.*,* individuals 1, 2, and 8), other samples had lower (individuals 3 and 5) or higher (individual 4) α diversity relative to immediate freezing (Fig. 1c).

We assessed the impact of different storage methods on the overall fecal microbiota composition at the OTU level using Bray-Curtis dissimilarity index (takes into account the relative abundances) and Jaccard distance (examines the presence/absence of taxa). The overall microbial community assessed by Bray-Curtis index was not significantly associated with the storage method both for abundant and rare OTUs (PERMANOVA, *p* > 0.05). In both cases, between individual differences were statistically significant (PERMANOVA, *p* < 0.001, R^2^ = 0.87 and 0.44 for abundant and rare OTUs, respectively, Fig. 1d). Similar results were also observed with Jaccard distance (not shown).

**Fig. 2:**
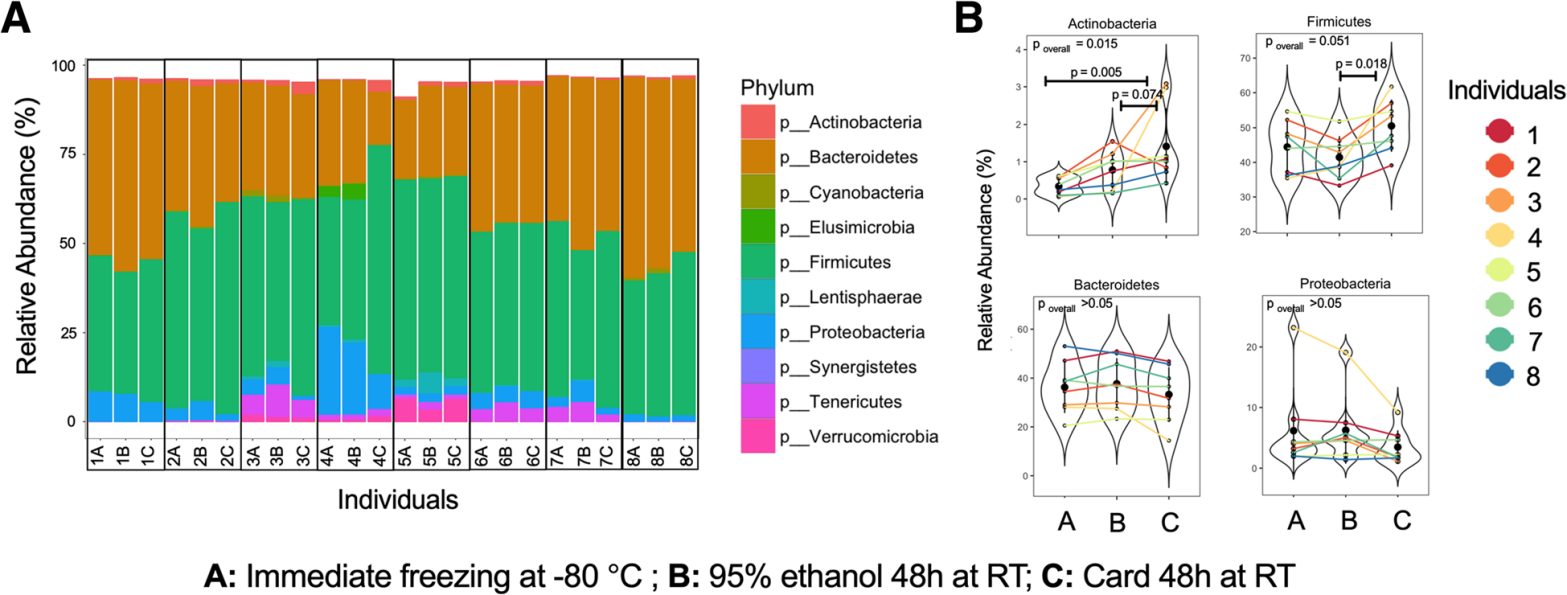
Comparison of relative abundances of gut microbiota profile at phylum level **a** across individuals, and **b** across methods

**Fig. 3:**
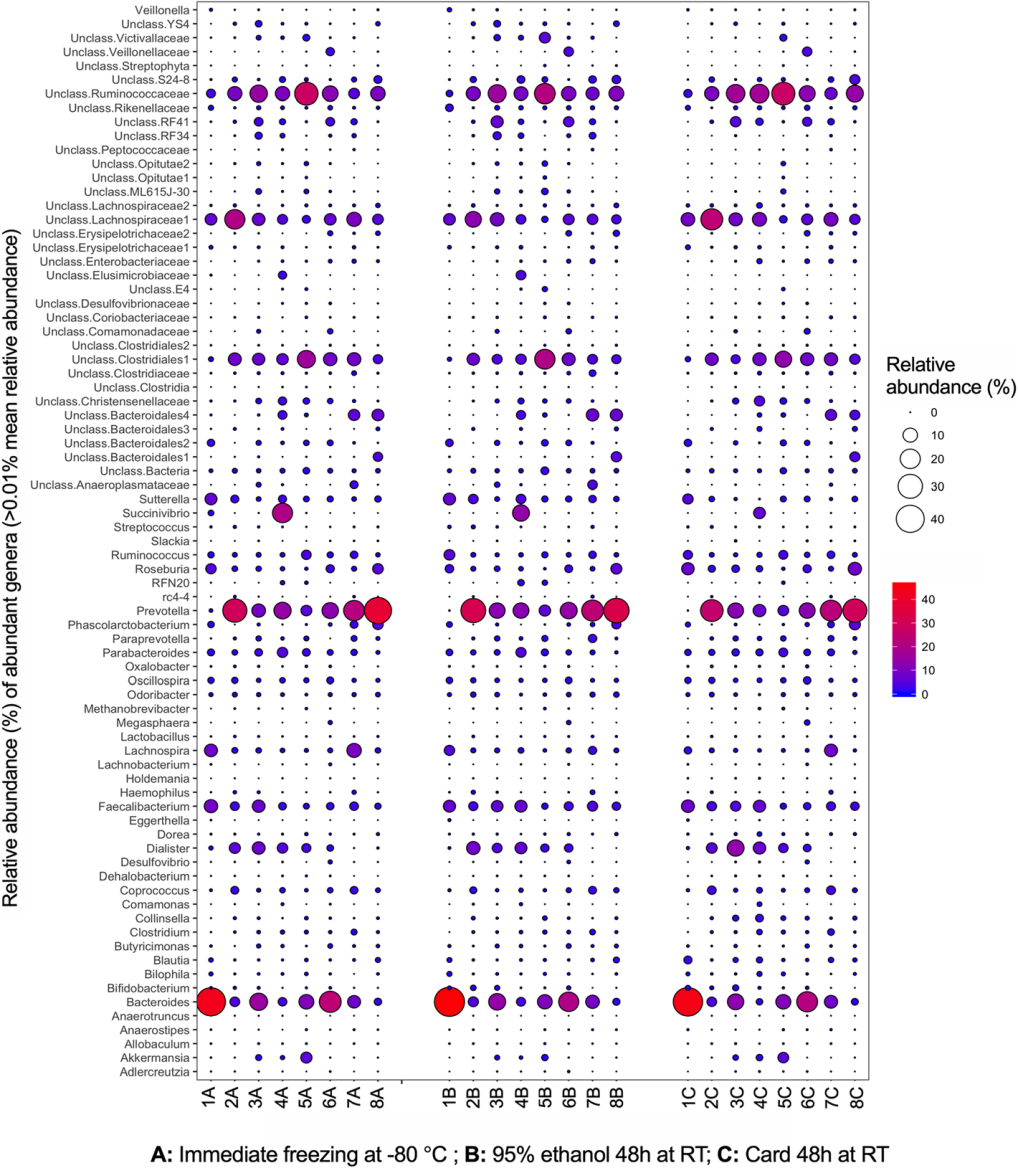
Comparison of relative abundances of abundant genera (> 0.01% mean relative abundance) across individuals and fecal storage methods

With the exception of Actinobacteria, fecal microbiota profiles at the phylum level were not significantly associated with the storage method. Indeed, inter-individual variation in the relative abundances of different phyla persisted across the different storage methods (Fig. 2a; ANOVA by individual not shown). Actinobacteria was significantly higher in samples collected on card compared to immediate freezing (1.6 ± 1.1% vs. 0.4 ± 0.2%, ANOVA, *p* = 0.005, Fig. 2b and Additional file 1: Figure S2). This observation was mainly driven by higher relative abundance of Actinobacteria in fecal samples stored on card in individuals 3 and 4 (Fig. 2a and b). The Actinobacteria phylum was dominated by *Bifidobacteriaceae* and *Coriobacteriaceae*. While relative abundances of *Bifidobacteriaceae* remained consistent between different storage methods, *Coriobacteriaceae* was enriched in samples stored on card (ANOVA, *p* = 0.002, Additional file 1: Figure S2). In addition, there was a trend in the difference of Firmicutes relative abundances across fecal storage conditions (ANOVA, *p* = 0.051). Although, there was not a significant difference between storage on card and immediately frozen samples, samples stored in ethanol had lower levels of Firmicutes compared to the card storage (Fig. 2b). Individual-level relative abundances at the family level remained consistent between methods for Bacteroidetes, Firmicutes, and Proteobacteria (Additional file 1: Figure S3). At the genus level, the most abundant taxa were *Bacteroides*, *Prevotella*, unclassified *Clostridiales*, unclassified *Lachnospiraceae*, and unclassified *Ruminococcaceae*. Storage method was not significantly associated with relative abundances of the abundant genera (> 0.01% mean relative abundance; Fig. 3). Patterns of microbiota taxonomic structure differences of the most abundant OTUs (> 1% mean relative abundance) did not show prominent differences when comparing storage in 95% ethanol and on card to immediate freezing (Additional file 1: Table S1 and Additional file 1: Table S2). The largest difference was observed for the genus *Succinivibrio* in one individual (individual 4). The relative abundance of this genera was 14% (3 folds) less abundant in samples stored on card relative to the sample preserved immediately at − 80 °C (6.4% vs. 20.1%; Additional file 1: Table S2).

## Discussion

In this study, we compared the combined effects of mock shipment and alternative collection and/or preservation methods (collection on card and preservation in 95% ethanol) to the gold standard of immediate freezing at − 80 °C on the observed microbial community structure in fecal samples. All methods delivered remarkably similar microbial community profiles, as indicated by the high similarity of replicate samples from the same individual. We observed that inter-individual variations were stronger than differences between different methods of fecal collection and storage in all microbiota features, including α and β diversity measures and relative abundances of dominant taxa at phylum and genus levels. In other words, the microbiota profiles remained consistent within individuals regardless of the storage method. This observation is in accordance with previous reports [13–17]. Strikingly, however, we observed that only 70% of abundant OTUs were shared among all different storage methods. This proportion was even less for rare OTUs, with 3% being shared, an indication of the stronger impact of the storage method on the observation of low abundance taxa within the fecal microbiota.

While the majority of studies focus on the correlation of results from different storage methods mainly using interclass correlation assessment [14–17], it is of interest that approximately two-thirds of the most abundant OTUs are shared across different methods in our study suggesting a less prominent impact of storage method on the most abundant taxa. However, only 3% of rare OTUs were detected in all three storage methods which could be attributed to the specifically higher inter-individual variability in less abundant taxa as well as higher sensitivity to the technical variation in the sequencing limit of detection. Our small sample size limits our power to examine the impact of storage conditions on the rare taxa in more details. Even though β diversity was not impacted by the storage method for the rare sub-community in our study, in cases where the research interest is in rare taxa, caution should be exercised in the choice of fecal storage.

Fecal collection methods are often in agreement with immediate freezing on the population level [13–17]. In accordance, we did not observe any differences in microbiota composition and structure on average. However, we observed individual differences specifically in microbial richness and relative abundances between different methods at individual level. For example, in one individual, richness was substantially low in the ethanol sample and higher in the card sample, relative to the frozen control. Conversely, in another individual, diversity was higher in ethanol and card compared to immediate freeze. Additionally, we observed higher relative abundance of Actinobacteria in samples stored on card in two individuals. Although, this observation could be attributed to different sample handling for these samples, the possibility of different behaviour of microbial communities in different storage conditions based on their original profile and internal dynamic cannot be excluded.

The main strength of population-based studies is a large sample size (usually on the scale of 50,000 to 200,000 samples), the availability of rich and comprehensive metadata, and longitudinal follow up [18]. Until recently, however, fecal samples were not routinely bio-banked in population-based cohorts. This trend is changing [19], leading to an interest in proper storage techniques. Samples collected on cards such as FOBT cards occupy considerably less freezer space and are preferable from a logistics perspective. However, other considerations need to be addressed, such as yield and quality of genomic DNA extracts. Under ideal conditions, where microbiota profiling from cards is consistent with that of immediate freezing, samples collected on these cards will still pose a technical challenge for the initial step of the DNA extraction by hindering automation. However, novel technical advances such as the use of specific punching tools may overcome such difficulty.

Although, we and others have observed consistent results of DNA-based microbiome analysis using alternative collection and storage methods, the performance of these approaches for other methods of gut microbiota interrogation such as metatrasncriptomics, metaproteomics, and metabolomics is not very commonly studied. One of the few available studies identified a subset of transcripts which were differentially abundant in alternative methods of sample storage [13]. Currently, DNA-based methods such as 16S rRNA gene sequencing is the method of choice for many projects as a first step of hypothesis generating which could then be followed up with more advanced techniques with higher taxonomic resolution. It could also be complemented with other readouts such as RNA and protein to be able to infer biological functions of the microbiota [20]. Although, fecal collection in ethanol was found to be suitable for metatrasncriptomics [13], preservation of RNA on card has not been investigated. However, card was shown to be suitable for fecal metabolomics [21]. Whether samples collected on card are also useful for bacterial culturomics and investigation of nonbacterial components of the gut microbiota (fungi, viruses, and protozoa) is another open question.

Similar to immediately frozen fecal sample, standardised protocols need to be developed for alternative methods of fecal collection and storage to minimize sources of lab-to-lab variation and to ensure repeatability and reproducibility [22–24]. Regardless of the collection method, attempts should be made to avoid repeated temperature fluctuation and to shorten the transportation time [25]. Results on the short-term storage of samples with alternative methods do not necessarily translate into unbiased microbiota results in long-term storage, which is often the case in cohort biobanks. In two small pilots, microbiome profile remained consistent as late as 10 years following storage on FOBT card [26, 27] suggesting the suitability of FOBT card (or similar products) for long-term cohort biobanking.

## Conclusion

In conclusion, acknowledging the small sample size in this pilot study, we provide evidence that microbiota profile of fecal samples collected in ethanol or on card and delayed storage at − 80 °C for up to 2 days is comparable to immediately frozen samples. Therefore, sample collection on card could potentially provide a practical alternative method of fecal biobanking in large-scale population-based cohorts. Although, the suitability of samples collected on card for microbiome studies other than 16S rRNA gene sequencing has not been confirmed, the cost- and space-effectiveness of this approach would be of importance to large-scale cohorts significantly facilitating the translational aspect of the microbiome research.

## Methods

### Study design

We performed a pilot study to assess the impact of different storage methods on fecal microbiota composition. Eight healthy volunteers provided self-collected fecal samples. To minimise variability, we recruited female research staff from Digestive Disease Research Institute with an age range of 25–40 years. Exclusion criteria included pregnancy or lactation at the time of sample collection, history of major gastrointestinal diseases including colorectal cancer or inflammatory bowel disease, and major gastrointestinal surgery. The study was approved by the Institutional Review Board of the Digestive Disease Research institute. Written informed consents were obtained from participants.

#### Sample collection

Individuals were instructed to collect the fecal samples using sterile fecal collection device (Pole Ideal Pars co., Iran). The samples were immediately transferred to the lab, where they were aliquoted. One aliquot was immediately stored at − 80 °C without preservative. Using a sterile swab, fecal material was transferred to tubes containing 95% ethanol and onto blood collection cards (Kawsar Biotech Company, Iran). Fecal samples in ethanol and on card were kept at room temperature (RT) for 48 h prior to storing at − 80 °C. The samples were stored for two months before DNA extraction.

##### Library construction and 16S rRNA gene sequencing

DNA extraction was performed with the QIAamp DNA Stool Mini Kit (Qiagen, Hilden, Germany) according to the manufacturer’s instruction. The V4 variable region of the microbial 16S rRNA gene was amplified with modified 515F/806R primers [28], and prepared for next-generation sequencing using a modified two-step targeted amplicon sequencing approach, as described previously [29]. Sequencing was performed using an Illumina MiSeq (Illumina, San Diego, CA, USA), with a V2 kit and paired-end 250 base reads at the Sequencing Core at the University of Illinois at Chicago. Raw fastq files for each sample were processed using the software package PEAR (Paired-end read merger) (v0.9.8) [30]. The merged fastq files were imported into the software package CLCGenomics Workbench v10.0 (CLC Bio, Aarhus, Denmark, Qiagen, Venlo, The Netherlands). Sequences without both the forward and reverse primers were discarded and subsequently, the primer sequences were removed. Sequences with low quality base calling scores (< 20) and insufficient length (< 250 bp) were discarded. Chimeric reads were filtered using USEARCH (v6.1) [31] within the open-source software QIIME (v1.8) environment [32]. Reads were clustered into OTUs using denovo OTU picking based on 97% similarity using UCLUST [31]. Representative sequences for each OTU were selected, and these sequences were assigned taxonomy using UCLUST and aligned to the 2013 release of the Greengenes reference database at 97% sequence similarity [33]. Demultiplexed sequencing data was deposited into the Sequence Read Archive of NCBI and can be accessed via accession number SRR8979312.

##### Microbial data pre-processing

Data analysis was conducted in R [34]. Initial preprocessing of the OTU table was conducted using the Phyloseq package [35]. Data was rarefied to the minimum 16,304 sequencing reads per sample. The numbers of sequencing reads of taxa were then relativized to the total sum of 16,304 without excluding rare taxa. Overall, 30,244 unique OTUs were detected. OTUs belonging to Archea, phylum Cyanobacteria, family of mitochondria, and class of chloroplast were excluded from the analysis resulting in 27,263 remaining OTUs.

##### Statistical analysis

The shared taxa were visualized using VennDiagram [36]. Alpha (α) diversity was assessed on the rarefied OTU table by the observed OTUs (richness) and inverse Simpson index (diversity). Association of α diversity with the storage method was assessed by one-way analysis of variance (ANOVA). Between-sample dissimilarity (β diversity) was assessed on Bray-Curtis dissimilarity and Jaccard distance indices for all and rare OTUs (< 0.01% mean relative abundance) by permutational ANOVA (PERMANOVA) using the vegan package [37]. To control for the compositional nature of the data, OTU counts were centre log-ratio transformed following zero-replacement [38, 39]. After this transformation, taxa relative abundances were compared at phylum and genus levels by ANOVA. Significant overall associations were further evaluated by post hoc pairwise comparison. The *P* values were corrected with Benjamini-Hochberg’s false discovery rate (FDR) method [40]. Corrected FDR *p* values of less than 0.05 were considered significant.

## Additional file

Additional file 1: **Table S1.** Percent difference in the relative abundances of fecal genera (> 1%) between immediate freezing at − 80 °C (A) and storage in 95% ethanol for 48 h at room temperature (B) in eight healthy volunteers. **Table S2.** Percent difference in the relative abundances of fecal genera (> 1%) between immediate freezing at − 80 °C (A) and storage on card stored for 48 h at room temperature (C) in eight healthy volunteers. **Figure S1.** Effect of storage condition on the fecal microbiota profile at species level for abundant species defined as having mean relative abundance of > 0.01% within each method. **Figure S2.** Comparison of relative abundances of members of Actinobacteria at family level. **Figure S3.** Comparison of relative abundances of gut microbiota profile at family level across individuals and methods. (PDF 1343 kb)

## Data Availability

The datasets generated and analysed during the current study are available in Sequence Read Archive of NCBI and can be accessed via accession number SRR8979312**.**

## Abbreviations

ANOVA: One-way analysis of variance
FDR: False discovery rate
FOBT: Fecal occult blood test
OTU: Operational taxonomic unit
PERMANOVA: Permutational ANOVA
RT: Room temperature
