# Supplementary material for: Assessment of the impact of different fecal storage protocols on the microbiota diversity and composition: a pilot study
Source: BMC Microbiol. 2019 Jun 28;19:145. doi: 10.1186/s12866-019-1519-2 (PMC6599303; doi:10.1186/s12866-019-1519-2)

Supplementary Material for "Assessment of the impact of different fecal storage protocols on the microbiota diversity and composition: a pilot study" (Moossavi et. al.)

Supplementary Tables & Figures

Table S1. Percent difference in the relative abundances of fecal genera (>1%) between immediate freezing at -80 °C (A) and storage in 95% ethanol for 48h at room temperature (B) in eight healthy volunteers.

Table S2. Percent difference in the relative abundances of fecal genera (>1%) between immediate freezing at -80 °C (A) and storage on card stored for 48h at room temperature (C) in eight healthy volunteers.

Figure S1. Effect of storage condition on the fecal microbiota profile at species level for abundant species defined as having mean relative abundance of > 0.01% within each method.

Figure S2. Comparison of relative abundances of members of Actinobacteria at family level.

Figure S3. Comparison of relative abundances of gut microbiota profile at family level across individuals and methods.

Table S1. Percent difference in the relative abundances of fecal genera (>1%) between immediate freezing at -80 °C (A) and storage in 95% ethanol for 48h at room temperature (B) in eight healthy volunteers.

OTU	Phylum	Family	Genus	1A-B	2A-B	3A-B	4A-B	5A-B	6A-B	7A-B	8A-B	Mean
OTU17728	Firmicutes	*Lachnospiraceae*	Unclass.	0.03	4.49	1.31	-0.03	0.00	-0.37	2.59	0.31	1.04
OTU7626	Proteobacteria	*Succinivibrionaceae*	*Succinivibrio*	1.27	0.00	0.01	6.39	0.01	-0.01	0.00	0.00	0.96
OTU27599	Proteobacteria	*Alcaligenaceae*	*Sutterella*	-0.34	0.00	-0.08	0.01	0.20	-0.04	0.08	0.52	0.04
OTU30617	Firmicutes	*Lachnospiraceae*	*Lachnospira*	1.74	0.27	0.00	-0.07	0.08	0.17	6.97	0.07	1.15
OTU37146	Firmicutes	*Ruminococcaceae*	*Faecalibacterium*	1.49	-0.38	1.26	-4.32	-0.29	-1.20	-1.40	-1.46	-0.79
OTU61854	Firmicutes	*Ruminococcaceae*	Unclass.	0.05	0.71	-0.37	0.55	1.73	0.72	0.29	0.46	0.52
OTU58911	Bacteroidetes	*Prevotellaceae*	*Prevotella*	0.05	0.00	-0.49	-0.13	0.00	0.00	0.00	0.49	-0.01
OTU47119	Bacteroidetes	*Bacteroidaceae*	*Bacteroides*	-0.23	0.01	0.31	0.00	0.00	1.56	0.00	0.00	0.21
OTU27602	Bacteroidetes	*Bacteroidaceae*	*Bacteroides*	0.68	0.08	0.49	0.02	0.22	1.58	-0.44	0.42	0.38
OTU61659	Bacteroidetes	*Bacteroidaceae*	*Bacteroides*	-1.86	-0.37	0.62	0.04	0.52	0.41	-0.32	0.20	-0.10
OTU55302	Bacteroidetes	*Bacteroidaceae*	*Bacteroides*	0.24	-0.16	0.06	-0.01	0.05	0.11	0.18	-0.09	0.05
OTU3931	Bacteroidetes	Unclass.*Bacteroidales*	Unclass.	0.07	0.00	0.13	-0.25	0.00	-0.01	-1.21	-0.24	-0.19
OTU63618	Bacteroidetes	*Prevotellaceae*	*Prevotella*	0.35	-0.85	-1.22	2.37	-0.90	-1.18	-1.99	4.95	0.19
OTU58361	Firmicutes	*Veillonellaceae*	*Dialister*	0.32	-2.86	1.96	-1.62	0.65	-1.45	0.00	0.11	-0.36
OTU63619	Firmicutes	*Lachnospiraceae*	*Roseburia*	1.52	0.23	-0.17	-0.08	0.11	0.34	0.17	-0.35	0.22
OTU72224	Firmicutes	*Lachnospiraceae*	Unclass.	0.08	0.78	-2.19	0.17	0.01	0.23	2.89	0.27	0.28

Table S2. Percent difference in the relative abundances of fecal genera (>1%) between immediate freezing at -80 °C (A) and storage on card stored for 48h at room temperature (C) in eight healthy volunteers.

OTU	Phylum	Family	Genus	1A-C	2A-C	3A-C	4A-C	5A-C	6A-C	7A-C	8A-C	Mean
OTU17728	Firmicutes	*Lachnospiraceae*	Unclass.	0.03	-3.15	2.22	0.61	0.00	0.86	0.94	0.31	0.23
OTU7626	Proteobacteria	*Succinivibrionaceae*	*Succinivibrio*	1.27	0.00	0.01	13.87	0.01	0.00	-0.01	0.00	1.89
OTU27599	Proteobacteria	*Alcaligenaceae*	*Sutterella*	1.52	-0.03	0.49	0.01	0.01	0.11	-0.04	0.48	0.32
OTU30617	Firmicutes	*Lachnospiraceae*	*Lachnospira*	2.41	0.21	0.00	0.10	0.01	0.21	1.83	0.07	0.60
OTU37146	Firmicutes	*Ruminococcaceae*	*Faecalibacterium*	0.81	-0.48	2.66	-4.48	-0.15	0.09	0.02	-1.59	-0.39
OTU61854	Firmicutes	*Ruminococcaceae*	Unclass.	0.05	-0.46	-0.78	-0.96	-0.32	-0.18	-0.27	-0.31	-0.41
OTU58911	Bacteroidetes	*Prevotellaceae*	*Prevotella*	0.05	0.00	-0.46	-0.94	0.00	0.00	-0.01	3.66	0.29
OTU47119	Bacteroidetes	*Bacteroidaceae*	*Bacteroides*	0.49	0.01	0.86	0.00	0.00	1.95	0.01	0.00	0.41
OTU27602	Bacteroidetes	*Bacteroidaceae*	*Bacteroides*	-0.35	0.12	0.75	1.15	-0.50	0.27	0.07	0.07	0.20
OTU61659	Bacteroidetes	*Bacteroidaceae*	*Bacteroides*	-1.89	-0.15	0.59	0.09	0.35	-0.04	-0.48	0.18	-0.17
OTU55302	Bacteroidetes	*Bacteroidaceae*	*Bacteroides*	0.81	-0.17	0.10	0.70	-0.01	0.33	0.02	0.04	0.23
OTU3931	Bacteroidetes	Unclass.*Bacteroidales*	Unclass.	0.07	0.00	0.13	2.80	0.00	0.00	0.18	2.08	0.66
OTU63618	Bacteroidetes	*Prevotellaceae*	*Prevotella*	0.35	2.88	-0.88	7.78	-0.10	0.74	-0.80	5.92	1.99
OTU58361	Firmicutes	*Veillonellaceae*	*Dialister*	0.25	0.42	-3.53	-2.39	-0.37	-0.70	0.00	0.11	-0.78
OTU63619	Firmicutes	*Lachnospiraceae*	*Roseburia*	-2.56	-0.97	-1.46	-1.03	-0.07	0.00	-0.04	-3.32	-1.18
OTU72224	Firmicutes	*Lachnospiraceae*	Unclass.	-0.05	-0.54	-2.72	-0.20	-0.03	-0.45	0.58	0.19	-0.40

Figure S1. Effect of storage condition on the fecal microbiota profile at species level for abundant species defined as having mean relative abundance of > 0.01% within each method.

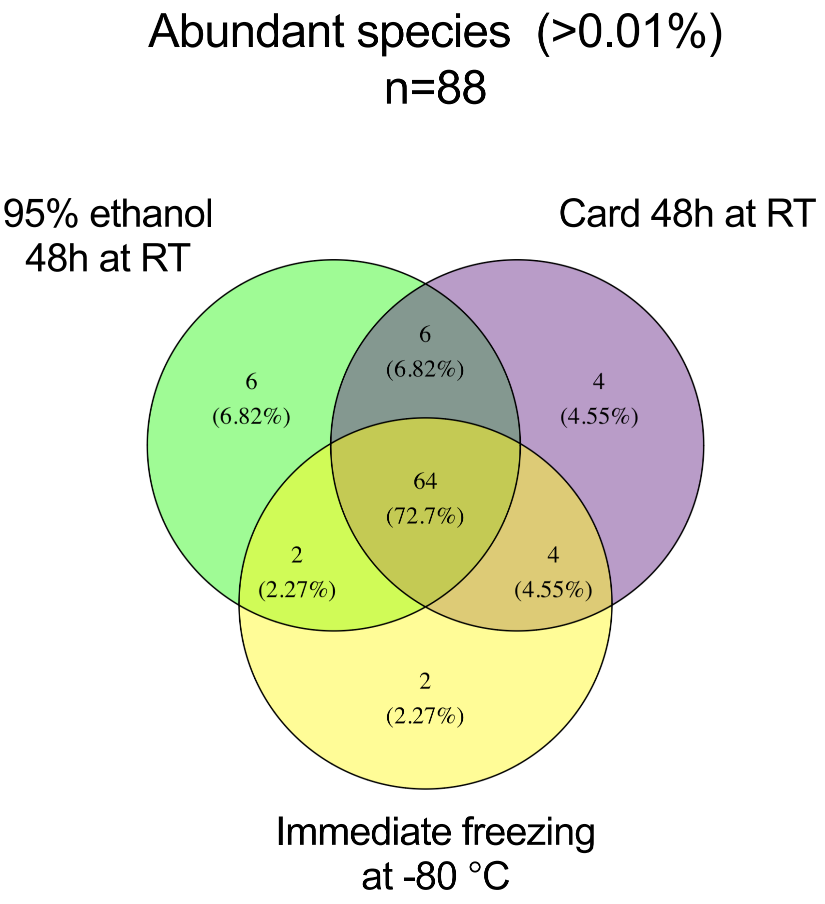

Figure S2. Comparison of relative abundances of members of Actinobacteria at family level. A) across individuals and B) across methods.

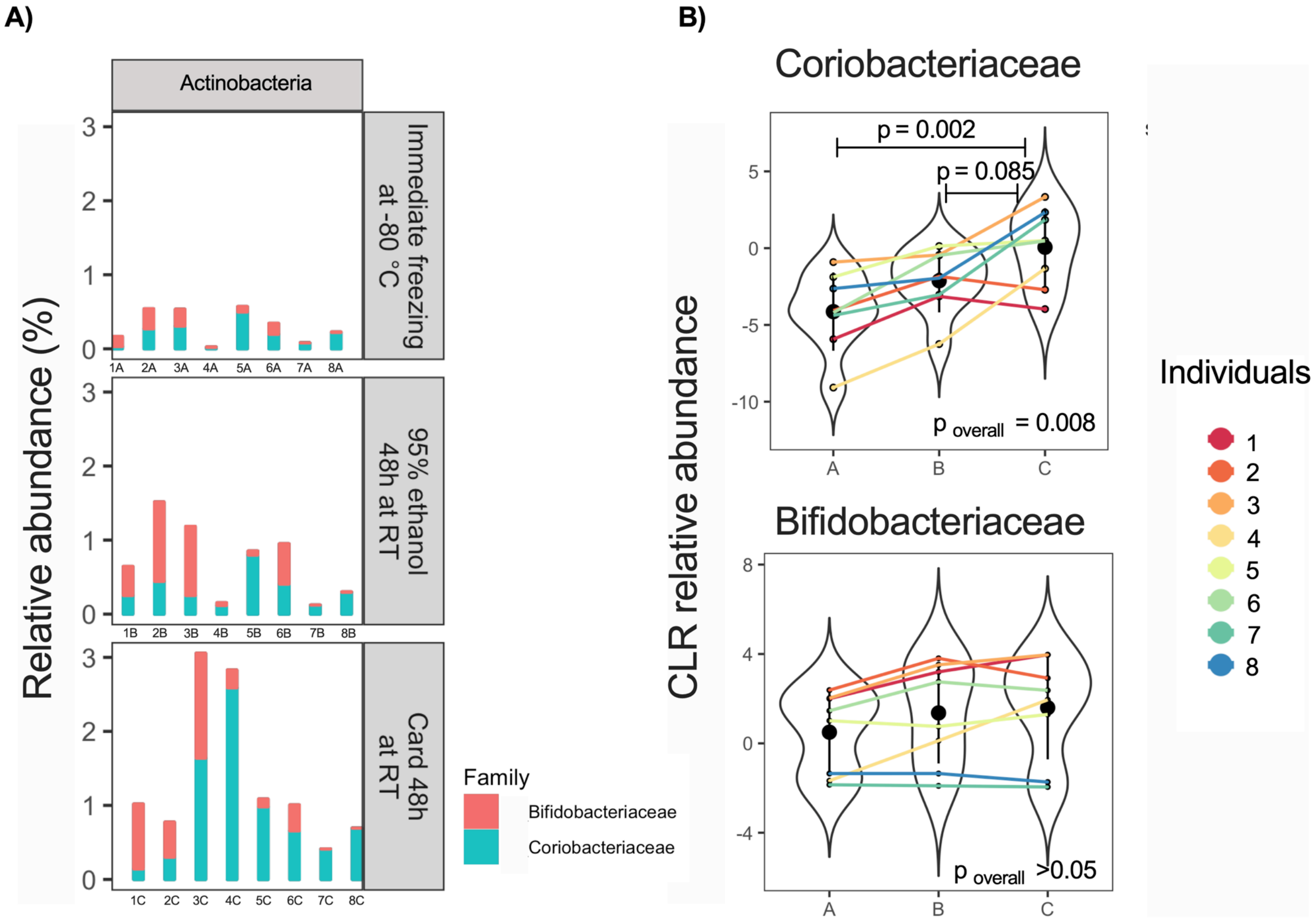

A: Immediate freezing at -80 °C ; **B:** 95% ethanol 48h at RT; **C:** Card 48h at RT

Figure S3. Comparison of relative abundances of gut microbiota profile at family level across individuals and methods.

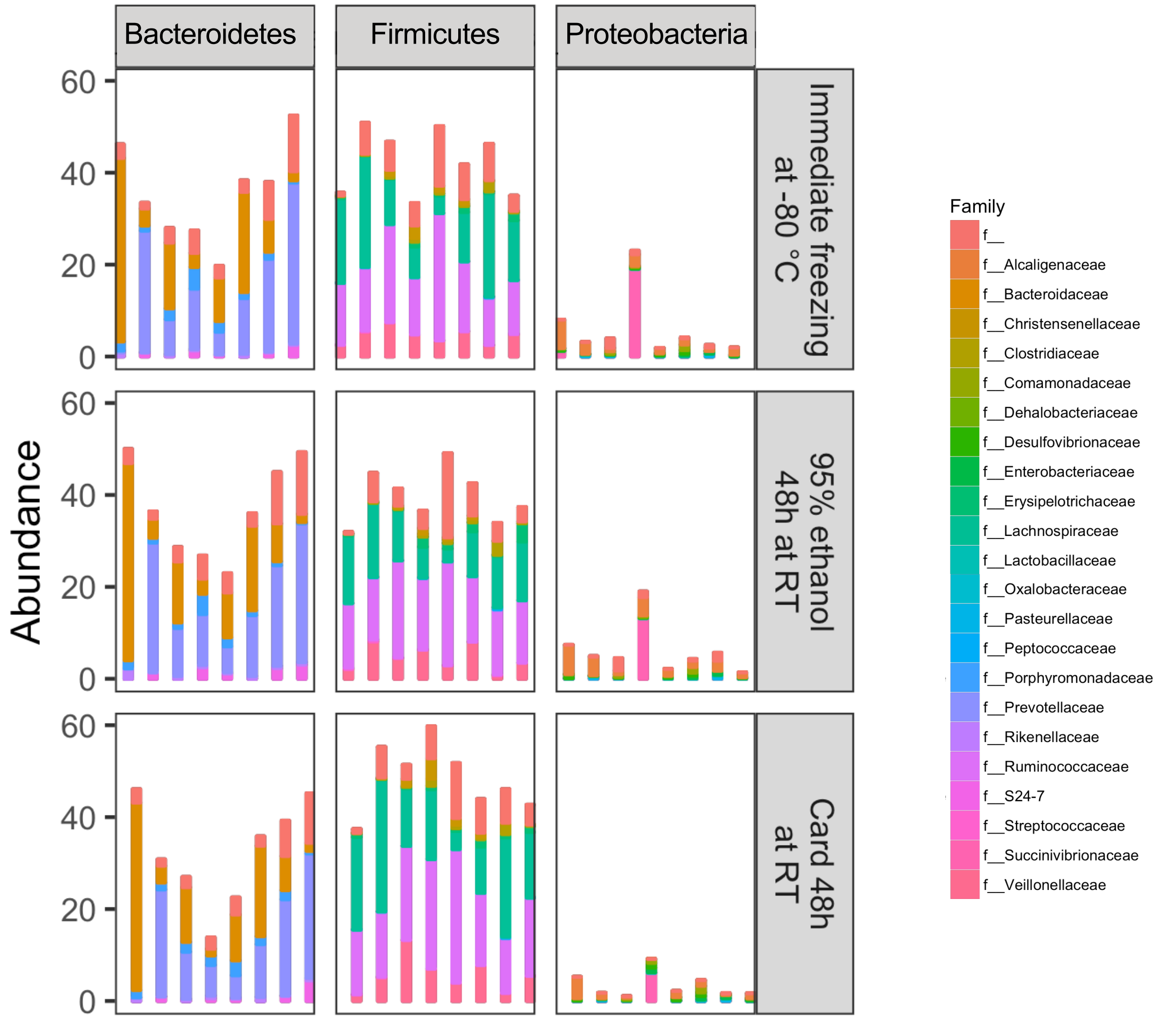

Supplement: Supplementary file 1 — Table S1. Percent difference in the relative abundances of fecal genera (> 1%) between immediate freezing at − 80 °C (A) and storage in 95% ethanol for 48 h at room temperature (B) in eight healthy volunteers. Table S2. Percent difference in the relative abundances of fecal genera (> 1%) between immediate freezing at − 80 °C (A) and storage on card stored for 48 h at room temperature (C) in eight healthy volunteers. Figure S1. Effect of storage condition on the fecal microbiota profile at species level for abundant species defined as having mean relative abundance of > 0.01% within each method. Figure S2. Comparison of relative abundances of members of Actinobacteria at family level. Figure S3. Comparison of relative abundances of gut microbiota profile at family level across individuals and methods. (PDF 1343 kb) [file 12866_2019_1519_MOESM1_ESM.pdf]
